# Endmember Learning with K-Means through SCD Model in Hyperspectral Scene Reconstructions

**DOI:** 10.3390/jimaging5110085

**Published:** 2019-11-15

**Authors:** Ayan Chatterjee, Peter W. T. Yuen

**Affiliations:** Centre for Electronic Warfare, Information and Cyber, Cranfield Defence and Security, Cranfield University, Defence Academy of the United Kingdom, Shrivenham SN6 8LA, UK

**Keywords:** sparse coding, dictionary learning, unmixing, hyperspectral scene reconstruction, hyperspectral, multispectral, k-means

## Abstract

This paper proposes a simple yet effective method for improving the efficiency of sparse coding dictionary learning (DL) with an implication of enhancing the ultimate usefulness of compressive sensing (CS) technology for practical applications, such as in hyperspectral imaging (HSI) scene reconstruction. CS is the technique which allows sparse signals to be decomposed into a sparse representation “a” of a dictionary $D_{u}$. The goodness of the learnt dictionary has direct impacts on the quality of the end results, e.g., in the HSI scene reconstructions. This paper proposes the construction of a concise and comprehensive dictionary by using the cluster centres of the input dataset, and then a greedy approach is adopted to learn all elements within this dictionary. The proposed method consists of an unsupervised clustering algorithm (K-Means), and it is then coupled with an advanced sparse coding dictionary (SCD) method such as the basis pursuit algorithm (orthogonal matching pursuit, OMP) for the dictionary learning. The effectiveness of the proposed K-Means Sparse Coding Dictionary (KMSCD) is illustrated through the reconstructions of several publicly available HSI scenes. The results have shown that the proposed KMSCD achieves ~40% greater accuracy, 5 times faster convergence and is twice as robust as that of the classic Spare Coding Dictionary (C-SCD) method that adopts random sampling of data for the dictionary learning. Over the five data sets that have been employed in this study, it is seen that the proposed KMSCD is capable of reconstructing these scenes with mean accuracies of approximately 20–500% better than all competing algorithms adopted in this work. Furthermore, the reconstruction efficiency of trace materials in the scene has been assessed: it is shown that the KMSCD is capable of recovering ~12% better than that of the C-SCD. These results suggest that the proposed DL using a simple clustering method for the construction of the dictionary has been shown to enhance the scene reconstruction substantially. When the proposed KMSCD is incorporated with the Fast non-negative orthogonal matching pursuit (FNNOMP) to constrain the maximum number of materials to coexist in a pixel to four, experiments have shown that it achieves approximately ten times better than that constrained by using the widely employed TMM algorithm. This may suggest that the proposed DL method using KMSCD and together with the FNNOMP will be more suitable to be the material allocation module of HSI scene simulators like the CameoSim package.

## 1. Introduction

Hyperspectral imagery (HSI) contains detailed spatial and spectral information of a natural scene. HSI has been traditionally deployed widely for surveillance and reconnaissance in agricultural, earth observations and defence/military applications [1]. In the latter case, particularly in counter-countermeasures applications, there are high demands on the knowledge about the detectability of targets when they are embedded in certain environments, for example, the assessment of the detectability of diseased plants in a field or the development of sophisticated camouflage materials for specific terrain and environment. In principle, this can be achieved through repeated costly and labour intensive experimental trials until the desire result is obtained. Alternatively, this can be accomplished through effective hyperspectral scene simulation technique, which is capable to reconstruct the scene and at the same time to “inject” foreign materials into the environment [2,3]. The ultimate usefulness of a scene simulator is its ability to simulate the scene faithfully, such that the spectral and spatial content of the simulation is as realistic as possible. This, in turn, will require knowledge of the material property in the scene, such as the types of plants and their optical characteristics, to be known precisely as the input of HSI simulator. In many cases, the detailed material properties of the scene are not known and typically only the broadband RGB, or the multispectral image (MSI) of the scene, is available [4,5]. One approach to solving the problem is to reconstruct the materials of the scene through a learned dictionary to deduce the endmember (EM) characteristics of the scene through an unmixing algorithm [6,7], Equation (1).

(1) $$y=D_{u}a+\epsilon$$

where “*y*” is the pixel in the scene, ϵ is the noise and $D_{u}$ is the EM dictionary. The learned EM is then applied to the MSI of the input image to allow the reconstruction of the HSI data [8,9]. This dictionary learning approach is commonly conceptualised through the convex optimisation of the linear inverse problem (LIP) with typical cost function “*J*” is shown in Equation (2).

(2) $$J=\min||y-D_{u}{a||}_{2}^{2}+{\gamma ||a||}_{1}$$

The dictionary set $D_{u}$ learns from the scene, and the abundance set “a” over the dictionary exhibits characteristic high sparsity over the $D_{u}$; γ is the parameter to adjust the balance between the two terms in Equation (2). To learn the $D_{u}$ for a specific data set, the normal approach is the selection of spectra from a comprehensive dictionary (also known as completed or overcompleted dictionary) [10], which consists of vast number of spectral database, then each of it is tested as according to Equation (2) to justify if it fits into the criteria. The process is repeated over the comprehensive dictionary until all the elements of $D_{u}$ is found or until all spectral database in the dictionary is exhausted. Previously, the comprehensive dictionary has been constructed from the pixel in the scene (called self-dictionary) [11], randomly selected from the scene [8], which sometimes in combination with spectral library data [9,12,13]. The quality of the scene reconstruction using the learned $D_{u}$ and “a” is critically dependent on how the $D_{u}$ is learned. Furthermore, most, if not all, of the existing dictionary learning algorithms are computationally not efficient and also lack of robustness, which imposes direct impacts on the quality of the HSI reconstruction negatively.

The focus of this paper is twofold: (i) To enhance the quality of HSI scene reconstruction through a robust dictionary learning method. (ii) To reduce the computational complexity of dictionary learning process. The paper reports a strategic dictionary learning through an unsupervised clustering method (K-Means [14,15]) as a preprocessing step in addition to an advanced basis pursuit algorithm (orthogonal matching pursuit (OMP)) for HSI scene reconstructions. The scene reconstruction capability of the proposed method is validated by using several publicly available data sets, which are then compared with that of other spectral unmixing techniques. The paper is structured as follows. Section 1 highlights the problem statement and outlines the main objective of the present work. In Section 2, we outline the background and shortcomings of previous work. Section 3 includes a description of the proposed algorithm. Section 4 outlines the data sets that have been utilised in this work and the scene reconstruction assessment metrics. Section 5 contains the experimental results obtained by the proposed method together with that of the competing algorithms and Section 6 contains the discussion and conclusions.

## 2. Prior Work in Dictionary Learning (DL)

There are two main approaches towards the solution of Equations (1) and (2): one is to find the purest element in the scene through searching for the convex cone of the spectral data (see [6,7] for an overview), and the other is through the optimisation of the sparsity of the abundance “a” [16] or using greedy algorithm for learning the dictionary $D_{u}$. Classical search methods that exploit the convex distribution property of data such as the vertex component analysis (VCA) [17], more recent algorithms like the minimum volume simplex analysis (MVSA) [18] and the Collaborative Nonnegative Matrix Factorization (CoNMF) [19] etc., have provided good solutions especially when relatively pure pixels are present in the scene. The VCA, MVSA and the CoNMF algorithms have been employed here as competing HSI reconstruction methods to compare with the proposed algorithm (see Section 3 below).

**Figure 1:**
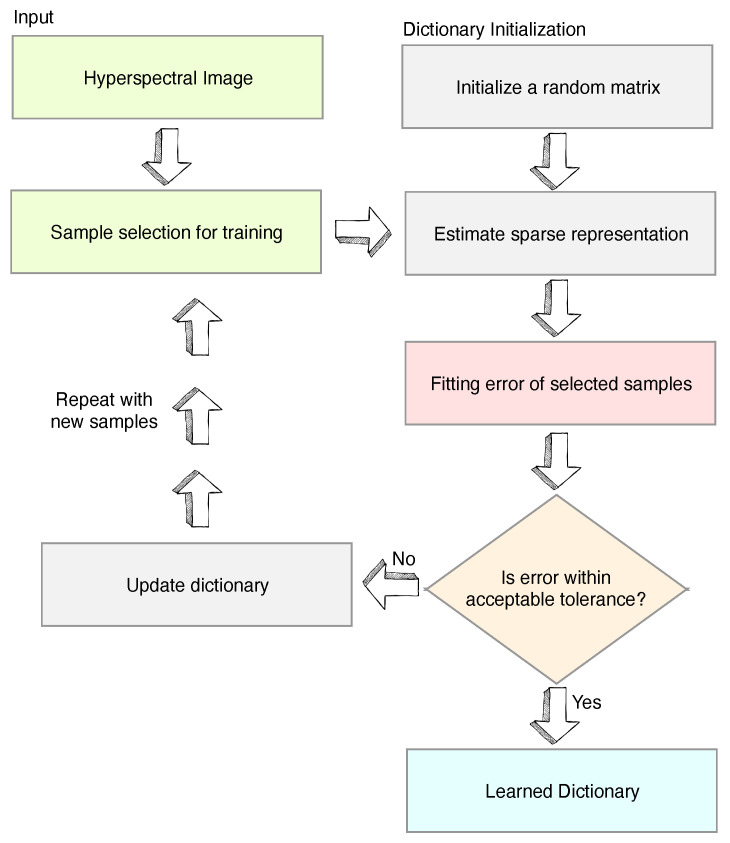
Flow diagram of the classic spare coding dictionary (C-SCD) algorithm.

Sparse Coding Dictionary (SCD) is a well-established method that decomposes the HSI to a linear collection of a few bases and a sparse matrix. The bases are called atoms, which have unity $\ell_{p}$ norm (generally $\ell_{2}$), and the sparse matrix is called representation. The group of atoms for a given scene is collectively called learned dictionary. SCD has an advantage when compared with the search method in that it is capable of finding $D_{u}$ even when pure pixels do not exist in the scene. SCD has a wide range of applications from super-resolution to classification [9,20,21]). The group of atoms for a given scene is called a dictionary. One such well-known SCD algorithm, proposed by Adam Charles et al. in [8], has demonstrated that the learnt basis $D_{u}$ highly resembles the real spectra, and it is capable of inferring HSI data from MSI images, when the “a” is explicitly constraint by a higher-order Laplacian prior in addition to their sparsity. This algorithm, shown in Figure 1, is referred to as classic Spare Coding Dictionary (C-SCD) in this paper. The C-SCD model has been shown capable of reconstructing the HSI scenes by using the dictionary that had been trained from the imagery acquired from another season of the same scene. It is seen from the objective function in Equation (2) that the learning of the SCD model is required to minimise both the $D_{u}$ and “a” at the same time. However, this cost function may not be jointly convex in the dictionary and the representation domains, which makes a global minimisation solution difficult. One solution to this issue is the adaptation of a variational approach in the C-SCD algorithm, which alternates the minimisation first with respected to the “a” for the given the current $D_{u}$, then a gradient descent over the elements in $D_{u}$ is taken for the given calculated “a” [8]. Furthermore, in the original C-SCD algorithm, the strategy for atoms selection during the learning process is not well defined which results in the reselection of already selected or rejected atoms in the later iterations, causing a severe slowdown of the algorithm convergence [22]. Although the C-SCD achieves a reasonable result, the complexity of the C-SCD learning is very high ≈O(N$K^{2}$), where N is the number of pixels in the scene, and K is the iteration number.

Recent advances in the dictionary learning (DL) has been made by using the greedy methods such as the orthogonal matching pursuit (OMP) [23], which finds the potential $D_{u}$ iteratively. New atoms are added whenever it has the largest absolute inner product with the residual, and subsequently the residual is updated, and the process is repeated. All the potential atoms found are grouped into a sub-dictionary, S, and new atoms are selected orthogonal to the subset S in the subsequent iterations. Enhancements such as the Simultaneous Orthogonal Matching Pursuit (SOMP), which estimates the residual of several atoms simultaneously in the same loop [11]; the constraint of the “a” non-negatively using non-negative least square (NNLS) to refine the selected representation in each iteration, together with the QR factorisation instead of pseudo-inverse of the dictionary to obtain the representation, have reduced the complexity of DL to O(NKlog(P)), where P is the inner loop count and P≪K [24]. This algorithm is also known as the fast non-negative orthogonal matching pursuit (FNNOMP).

To learn EMs with the C-SCD model, two fundamental changes were necessary [16]. The $\ell_{p}$ norm constraint from atoms is removed because real EMs often do not hold such constraint. Moreover, “a” is constrained for sum-to-one abundance as per the linear mixing model (LMM). Two approaches typically enforce the abundance condition. Fully constrained least squares (FCLS) for non-negative and sum-to-one abundance is enforced through two Lagrange multipliers, one for positive constraint with Karush–Kuhn–Tucker condition and the another for sum-to-one. Alternatively, by using one Lagrange multiplier to maintain the positive condition and the sum-to-one condition is concatenated with ones vector in the input signals and the EMs. The latter method is more favourable in computation time as it does not need the second Lagrange multiplier. More information about this approach is in Chang’s paper [25], which covers an overview of FCLS and other linear spectral mixture analysis methods.

## 3. Proposed Algorithm for Dictionary Learning (DL)

The objective of this paper is to propose a robust and efficient dictionary learning (DL) technique, which enhances HSI scene reconstruction with the given MSI or HSI data sets as inputs. The proposed technique reduces the computational complexity of the DL algorithm, and it has the capacity of achieving better spectral reconstruction not only for the background pixels (i.e., majority materials in the scene), but also the recovery of the minority pixels (i.e., trace materials in the scene) from the scene. This is in contrast to the C-SCD algorithm in [8], which learns the dictionary in a suboptimal way by randomly selecting pixels from the scene during the learning cycle, thus resulting in very variable performances even when the same scene is run repeatedly.

Two algorithms have been proposed here, and both have adopted a clustering algorithm (K-Means) to obtain a more representative spectral structure of the scene. The number of clusters ($J_{c}$) have been designed to be a few times more than the intrinsic dimension (i.e., the number of EMs in the scene) to include very small minority pixels in the scene to be clustered. These cluster centres set Ci, is then formulated as the dictionary of the scene for the algorithm to learn. This is in great contrast to the conventional DL algorithms, which construct their comprehensive dictionaries using all pixels in the scene. The number of the pixels (N) in the HSI scene is typically in the order of hundreds of thousands, comparing with the limited number of elements ($J_{c}$) in Ci in the order of approximately one-hundred pixels, the learning loop which utilises the Ci set will immediately cut the computational complexity down by several orders of magnitudes.

The first algorithm is the modification of the C-SCD by substituting its overcompleted dictionary by the Ci pixel set; the algorithm is then forced to select every member of the Ci to obtain the learned dictionary rather than through random selections. The mini-code of this K-Means SCD algorithm (KMSCD) is shown in Algorithm 1, and the complexity of the KMSCD is ≈O($J_{c}K^{2}$), where ($J_{c}$) is the number of clusters. The second algorithm (Algorithm 2) is designed for applications where a limited number of mixtures ($N_{mp}$) are allowed per pixel. In most HSI scene simulators, a limit of up to three or four mixtures per pixel [26] is allowed for the construction of the pixel texture. This Algorithm 2 first learns the $D_{u}$ using the KMSCD, but the abundance “a” is subsequently constrained through the FNNOMP, which limits the maximum mixture per pixel to be four different materials (EMs). Note that, in Algorithm 1, there is no limit on the number of mixtures per pixel.

**Algorithm 1** Proposed K-Means SCD (KMSCD) algorithm.1:**Import** HSI Image as ’Y’, number of EMs ’m’, user-defined step size ’s’ and step-size decay ’d’, and maximum number of iterations as ’M’2:Classify ’Y’ into ’$J_{c}$’ classes, where ’$J_{c}$’ is also number of samples per iteration3:**Initialise** EM dictionary as $D_{u}$ as ’m’ random numbers4:**for**k = 1 to K**do**                        ▹ Iterate till convergence5:    Select $Y_{k}$ samples, one from each ’$J_{c}$’ class6:    $a_{k}$ = $\min||Y_{k}-D_{u}a_{k}{\left|\right|}_{p},\forall a_{x}\geq 0$ and $\left|\right|a_{k}{\left|\right|}_{1}=1$    ▹ Infer positive sum-to-one abundance7:    $D_{k}$ = $\left(Y_{k}-D_{u}\;\cdot \;a_{k}\right)\times a_{k}$                   ▹ EM update estimation8:    $D_{u}\overset{}{\leftarrow }D_{u}+s\times D_{k}$                     ▹ Update EM dictionary9:    s = s × d                              ▹ Update step-size
User-defined estimates like step size of 0.01 and decay of 0.99998, and non-negative least squares function to infer coefficients remain unchanged from [8] for a like-for-like comparison. An inner loop is required if more than one sample ’$Y_{k}$’ is selected per iteration to estimate “a”.

**Algorithm 2** Proposed for scene simulators: KMSCD+FNNOMP.
1:**Import** HSI Image as ’Y’, number of EMs ’m’, maximum number of EM materials per pixel ’$N_{mp}$’, user-defined step size ’s’ and step-size decay ’d’, and maximum number of iterations as ’M’2:Classify ’Y’ into ’$J_{c}$’ classes, where ’$J_{c}$’ is also number of samples per iteration3:**Initialise** EM dictionary as $D_{u}$ as ’m’ random numbers4:**for**k = 1 to K**do**                        ▹ Iterate till convergence5:    Select $Y_{k}$ samples, one from each ’$J_{c}$’ class6:    $a_{x}$ = FNNOMP($D_{u}$, $Y_{k}$, $N_{mp}$), $\forall ||a_{i}{\left|\right|}_{1}=1$7:    $D_{k}$ = $\left(Y_{k}-D_{u}\;\cdot \;a_{k}\right)\times a_{k}$8:    $D_{u}\overset{}{\leftarrow }D_{u}+s\times D_{k}$9:    s = s × d


## 4. Data Sets and Accuracy of Scene Reconstruction Assessments

Herein, we use a scene from the Selene dataset [27]. The scene “Selene H23 VNIR” was acquired by HySpex VNIR-1600 sensor at Porton Down range (Long 51°8′19.7″ N Lat 1°39′16.9″ W to 51°7′41.7″ N 1°40′8.5″ W) on 12 August 2014 BST 12:00:04. Natural materials like grass, soil and tree cover over 95% of Selene scene, and artificial materials such as ground markers, path, concrete, building and coloured panels cover the remaining scene. Furthermore, the scene “Selene H23 Dual” was co-registered from the HySpex VNIR-1600 and SWIR-384 data sets, have been used as the two main data sets in this paper. The Dual image has a ground sampling distance (GSD) of 70 × 70 cm compared to the VNIR with a GSD of 17 × 34 cm. QUAC [28] was applied to the raw data using ENVI software with a generic sensor to obtain the reflectance of this dataset. Apart from Selene, the publicly available “Paso Robles-Monterey” (AVIRIS dataset flight name: f150615t01p00r11) which is a high altitude AVIRIS imagery consisting of vegetation, highway, and cities, and three “Virginia City” images (accessed from https://www.spectir.com/contact#free-data-samples), which are images of a mountainous region, have also been employed for experimental validation of the proposed algorithm. Table 1 summarises the dimensions of the scenes.

All experimental runs were performed under the same configurations: (1) The DL has been performed in two cases: one is using the whole scene to learn, which means the whole data set has been utilised for training the EM, and the other is using halve of the scene to train the dictionary. (2) All experimental runs were repeated 5 times and the averaged reconstruction accuracies were then obtained. (3) All experiments using the proposed and the C-SCD method were performed by a i7-6700K Quad core CPU, and the typical convergence time for the C-SCD runs were ~5 times longer than that of the KMSCD

We analysed the reconstruction error, or fitting error, with two distance metric—Manhattan distance (MD) and percentage differential $\ell_{1}$ norm error (DL1NE). Sum of absolute point to point distance difference, or MD, demonstrates how much error there is in terms of magnitude difference. MD with a total of “B” bands is shown in Equation (3). However, we choose a second metric, DL1NE, to avoid suppressing errors of low reflective materials. For instance, a DL1NE of 200% demonstrates that the reconstructed pixel is three times in area for the same ground truth pixel. DL1NE is given by Equation (4).

(3) $$\mathrm{MD}\;=\;\ell_{1}\mathrm{norm}\;=\;\sum_{b=1}^{B}|\mathrm{ground}\;\mathrm{truth}\;-\;\mathrm{reconstructed}|$$

(4) $$\mathrm{DL}1\mathrm{NE}\;=\;|\frac{\ell_{1}\;\mathrm{norm}\;\mathrm{of}\;\mathrm{ground}\;\mathrm{truth}\;\mathrm{pixel}\;-\;\ell_{1}\mathrm{norm}\;\mathrm{of}\;\mathrm{reconstructed}\;\mathrm{pixel}}{\ell_{1}\;\mathrm{norm}\;\mathrm{of}\;\mathrm{ground}\;\mathrm{truth}\;\mathrm{pixel}}|\times 100$$

The MD is usually normalised with respected to the number of spectral bands, especially when it is used to compare the goodness of the algorithm for several data sets that have different number of spectral bands. Both spectral distance error metric gives indication of the goodness of the reconstruction only for the background materials, which are abundant in the scene; however, these methods are not sufficiently sensitive enough to quantify the errors of very trace materials, e.g., the very small number of artificial materials typically few % in the natural scene (e.g., the colour panels in the Selene data set). In this case, a target detection algorithm known as the adaptive cosine estimator (ACE) [29,30] has been adopted for testing the ability of the reconstruction to recover trace targets in the scene. ACE is shown in Equation (5) for *i*th pixel, where *s* is the known target, $x_{i}$ is the *i*th pixel in question, $\bar{x}$ the mean of the whole scene, *C* is the pseudoinverse covariance matrix and ${}^{T}$ represents transpose.

(5) $${\mathrm{ACE}}_{i}=\frac{{\left({\left(s-\bar{x}\right)}^{T}C\left(x_{i}-\bar{x}\right)\right)}^{2}}{\left({\left(s-\bar{x}\right)}^{T}C\left(s-\bar{x}\right)\right)\left({\left(x_{i}-\bar{x}\right)}^{T}C\left(x_{i}-\bar{x}\right)\right)}$$

**Figure 2:**
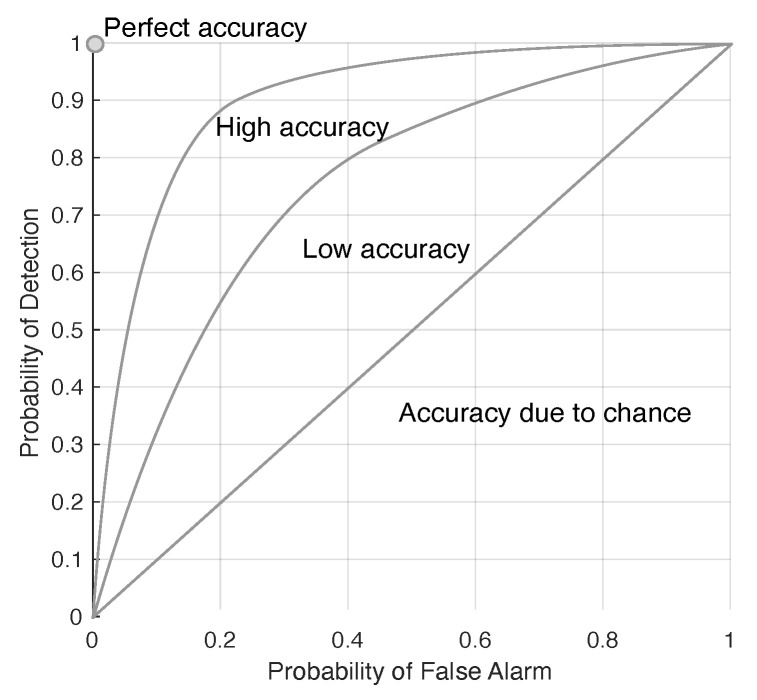
Illustrating the implication of the receiver operating characteristics (ROC) in target detection.

The detectability of the target is presented in a receiver operating characteristics (ROC), which plots the positive–positive versus the positive–negative, and an example of the ROC is illustrated in Figure 2. The locations of targets (i.e., the target map) is noted from the ground truth data set (i.e., the Selene ground truth data in this paper). The ROC of the target is constructed by using the scores given by ACE detection of the target in the reconstructed scene, and it is then constructed using the target map information to indicate the faithfulness of the detection.

## 5. Results

### 5.1. Feasibility of K-Means Clustering for Multispectral Data Set

**Figure 3:**
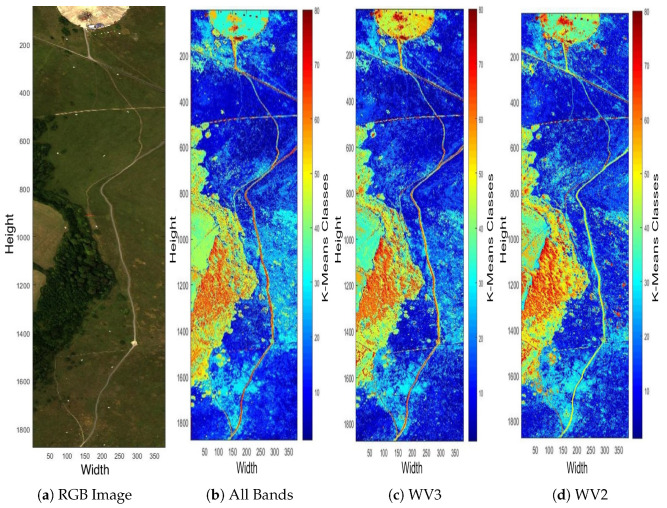
False-colour classification maps displayed with all 448 bands (**b**), 16 centre wavelengths of the WV3 sensor (**c**) and 8 centre wavelengths of the WV2 sensor (**d**) classified by K-Means for H23 Dual scene into 80 classes with MATLAB’s default maximum of 100 iterations. The figures show >99% of classification similarities despite of the very small number of spectral bands (8 bands) that has been utilised in (**d**).

As mentioned previously, one objective of this paper is to achieve an efficient HSI scene reconstruction given by a multispectral image (MSI) as input. The proposed method involves a preprocessing clustering method to extract the spectral characteristic of the scene. This section is attempted to verify how robust is the clustering when the input data contains only a few <10 spectral bands. Figure 3 depicts the false-colour maps of the Selene Dual scene clustered by K-Means into arbitrarily selected 80 centres over 100 iterations. The input data sets consist of (i) all 448 hyperspectral bands, (ii) 16 WorldView-3 (WV3) centred wavelengths and (iii) 8 WorldView-2 (WV2) centred wavelengths. The colour of pixels in the figure represents the classes that they are in and the assignment of the colours is random, meaning that the colour of *i*th class in all three cases can be different. The figure highlights how well the grass, tree, soil, artificial materials, etc. are clustered over the very different number of spectral bands in these three data sets. The classified patterns are shown in Figure 3 and exhibit ~99% similarity over the three results, indicating that the clustering is robust against the number of spectral bands of the input MSI data demonstrating the practicability of the KMSCD for the reconstruction of HSI from the MSI imagery.

### 5.2. C-SCD vs. KMSCD: Reconstruction of Background Pixels

#### 5.2.1. Robustness of C-SCD and the Proposed KMSCD

**Figure 4:**
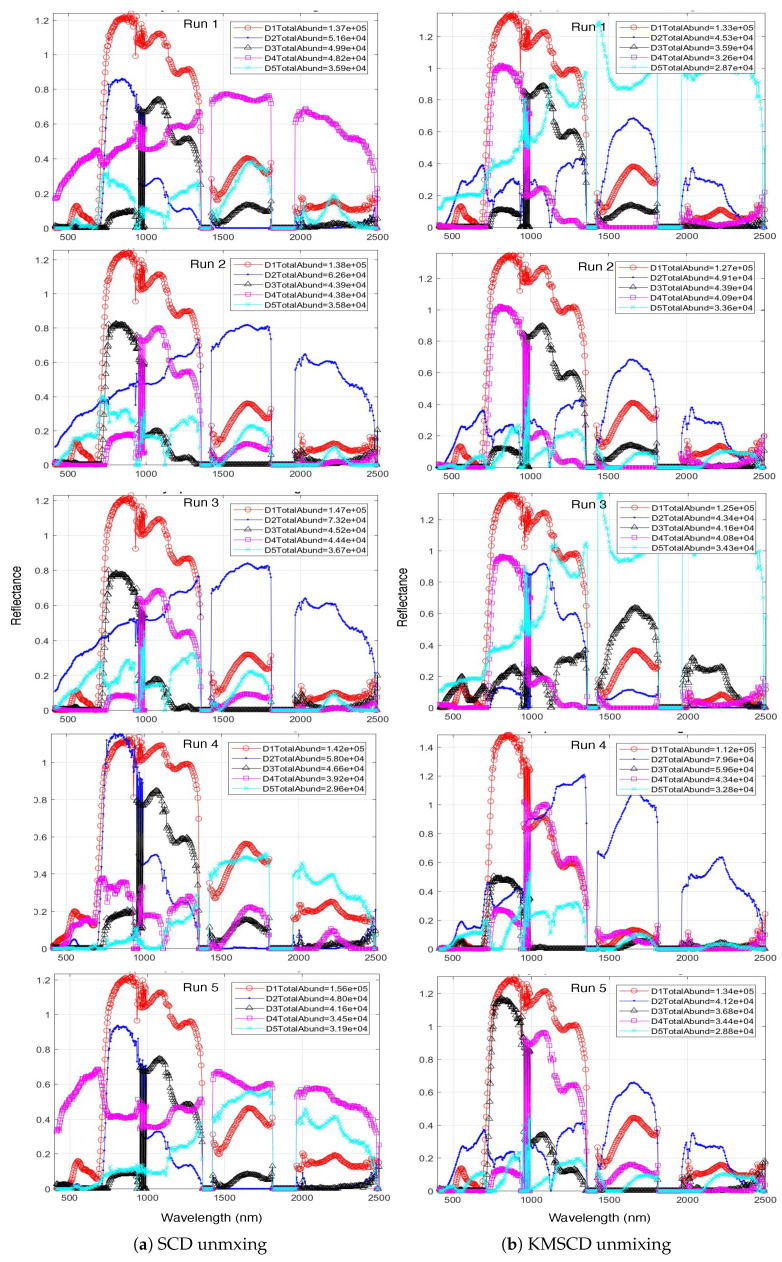
Most abundant endmembers (EMs) for the for five runs with 40 EMs between (**a**) SCD-unmixing with random sample selection and (**b**) the proposed K-Means SCD algorithm (KMSCD) unmixing.

This section aims to illustrate the robustness of the dictionary learning (DL) in C-SCD [8] and to compare this with the proposed KMSCD method (Algorithm 1). The robustness can be assessed by checking whether the most abundant EM in the scene can be reproducibility found over repeated runs of both algorithms. In both cases, the experiments were run using the whole data set for DL and the number of the K-Means cluster ($J_{c}$) set to the number of samples selected per iteration, which was 200 in C-SCD paper [8]. Figure 4 depicts the spectra of the first five most abundant materials in the reconstructed Selene Dual data set over five repeated runs obtained by the C-SCD and the KMSCD. The colour of the line plot is fixed as according to the order of the abundances, e.g., the red and the blue plots indicate the most and second most abundant materials in the reconstructed scene, respectively. Both methods manage to find the most abundant materials (in red plot) in four out of the five runs (i.e., they fail to find the most abundant materials correctly in the fourth run) with the total abundance of this material in the order of ~$1.4\times 10^{5}$, which is roughly ~20% of the scene. The scene contains 1876 × 380 ~$7.1\times 10^{5}$ pixels. However, the second most abundant material (in blue plot) that has been found by the C-SCD exhibit two quite distinct EMs with total abundances ranging between $5\times 10^{4}$ to $7\times 10^{4}$ respectively; whereas the KMSCD gives three out of five runs the same EM with abundances of ~$4.4\times 10^{4}$, which amounts to ~6% of the scene. This result may thus suggest that the proposed KMSCD performs slightly more robust than that of the C-SCD method.

**Figure 5:**
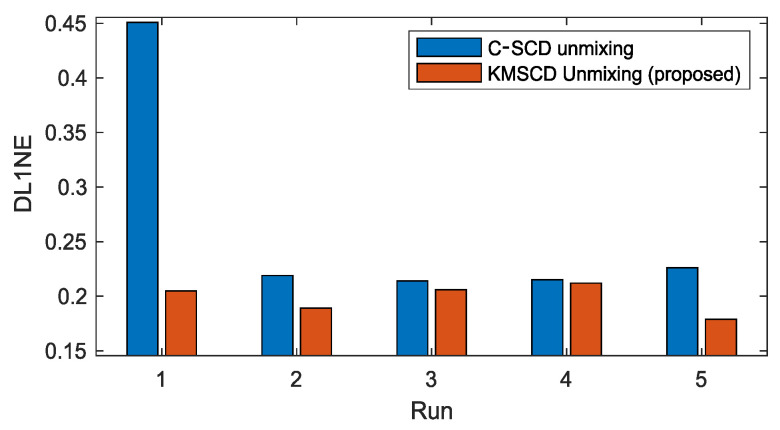
Plots the mean of the differential L1 norm (DL1NE) of the 5 repeated runs of the Selene Dual scene reconstruction performed by the C-SCD and the proposed KMSCD DL learning algorithms. The STD of the DL1NE processed by the C-SCD is almost double of that processed by the proposed method over the 5 experimental runs, further demonstrating the superior performance of the proposed KMSCD algorithm.

The robustness of the C-SCD and the KMSCD algorithms is further examined through their reproducibility of scene reconstructions over the repeated five runs. Figure 5 plots the mean of the DL1NE error given by the C-SCD and KMSCD algorithms. Over the five runs, the mean errors and standard deviations (std) of the C-SCD and KMSCD are, respectively, 0.32% ± 0.057% and 0.24% ± 0.027%. The STD of the KMSCD over the five runs exhibit roughly halve of that by C-SCD, further showing the much better robustness of the proposed KMSCD method in comparison to the C-SCD.

#### 5.2.2. Accuracy of C-SCD and KMSCD: Background Pixels

**Figure 6:**
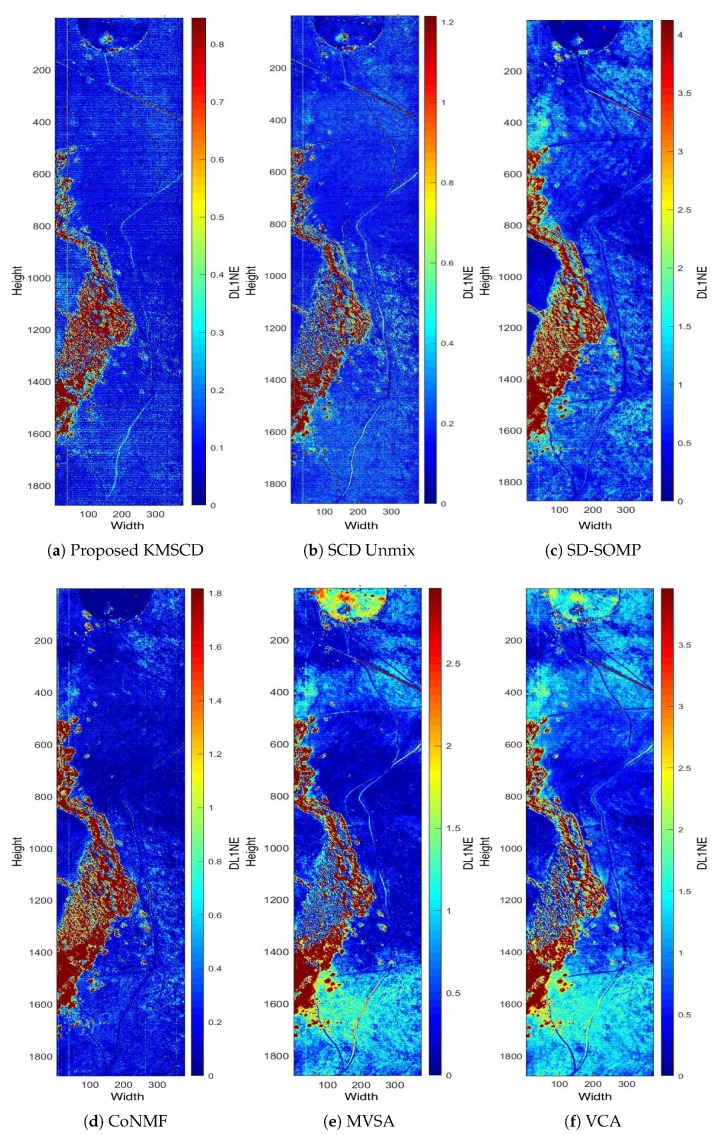
False-colour map of DL1NE of different methods on H23 Dual scene when trained from the first 1000 lines, whose mean error is mentioned in Table 2. Each of the error maps has been presented in various scales of [0 to 3 × mean(DL1NE)], such that the consistency of the reconstruction performance over the entire scene among all methods can be examined.

This section presents the main results of this paper, and the first focus is to verify the effectiveness of the scene reconstruction for the background materials in the scene. Subsequently, the ability of the DL algorithms to reconstruct trace materials is presented in the next section (Section 5.3). Figure 6 shows the DL1NE (i.e., the differential $\ell_{1}$-norm of the reconstructed vector w.r.t. that of the ground truth) false-colour map of the Selene Dual scene reconstructed by the proposed KMSCD (i.e., the Algorithm 1) and also by other competing algorithms. The data input in all cases was the Selene Dual 448 band data set and the sizes of the dictionary to be learnt were fixed at M = 50. In the classification, a cluster is ideally assumed to be a candidate material on its own. We substituted random sample selection and forced KMSCD to select one sample from each cluster. The DL for all methods was performed using the first 1000 lines (approximately half of the scene). The result of the reconstruction presented in Figure 6 is in false colour, and, to visualise the consistency of the reconstruction performance over the entire scene among all competing methods, all results have been presented to show errors up to a maximum of 3 times of the mean of the DL1NE error over the entire scene. For example, the colour patterns in Figure 6a,b are very similar: both exhibit high errors (in red) in the tree areas, whereas good reconstructions (i.e., blue colour) are seen over the glass and the concrete slab in the scene. The differences between these two, are that the proposed KMSCD result (Figure 6a) gives a mean of the DL1NE of 0.28% over the entire scene, which is ~40% better of the reconstruction accuracy than that of the C-SCD (Figure 6b). These two figures also highlight the more superiority in the proposed KMSCD for the reconstruction of the most abundant material in the scene (i.e., the grass) over the C-SCD method: the whole scene apart from the tree area are all in very low error <0.2% (in blue colour), and it can only be seen from the KMSCD result. Over the five data sets employed in this study, it is seen from Table 2 that the proposed KMSCD is capable of reconstructing these scenes with the lowest error over all competing algorithms that have been adopted in this research. Similar to Table 3, Table 4 tabulates the mean of the MD error of the entire scene for various data sets that have been employed in this work. Table 4 is the same as Table 3, but the mean(MD/band) has been tabulated so to allow direct comparisons between data sets that may have a differing number of spectral bands.

It is seen from these Tables that the smallest errors (DL1NE and MD) over all five data sets that were utilised in this study is achieved by the proposed KMSCD method, which exhibits performances that are approximately between 20–500% better than all competing algorithms. The enhancement figures that are tabulated at the bottom line of Table 2, Table 3 and Table 4, which utilise two different error metrics (i.e., the DL1NE and the MD) for the assessment, are found to be very close to linear dependency relationship (not shown here for brevity), implying that the accuracy assessments are highly consistent. The best performances are denoted in bold.

### 5.3. Reconstruction of Trace Materials in the Scene

#### 5.3.1. C-SCD vs. KMSCD

**Figure 7:**
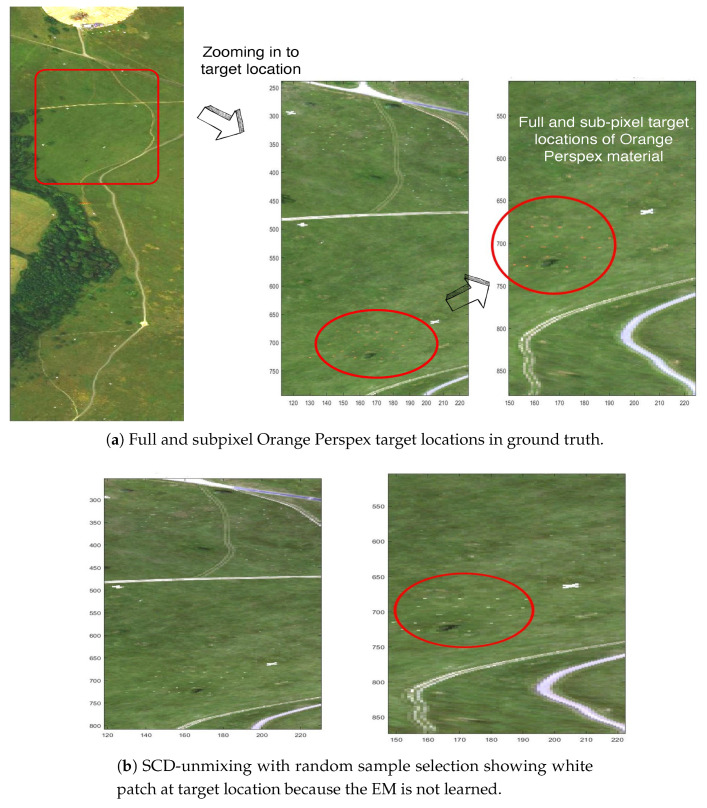

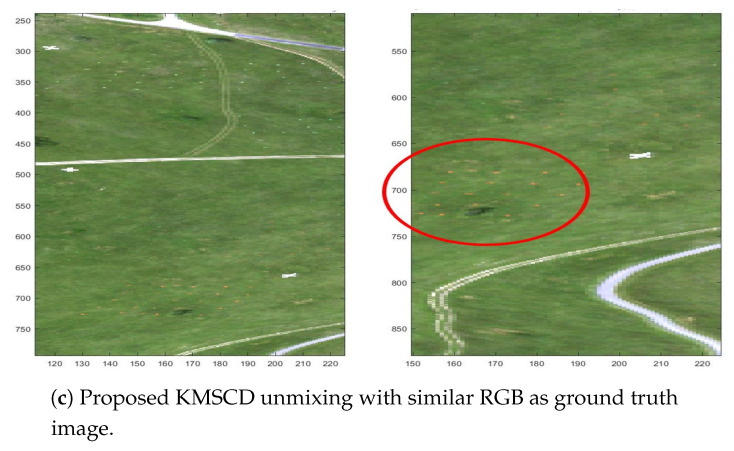
RGB image of the Selene Dual data set to show the location of the trace target materials and the ability of DL algorithms to recover them.

This section describes the abilities of the DL algorithms, precisely, the C-SCD and the proposed KMSCD algorithms, for the reconstruction of trace materials in the scene. There is a small amount (~1 of whole scene) of full-pixel (i.e., material occupancy = 1) artificial materials embedded in the Selene scene, and their presence in the scene are depicted in the RGB image as shown in Figure 7a. A number of small Orange Perspex panels with dimensions of approximately 40 × 40 cm, deployed in the scene as targets, are used here to testify the ability of the DL algorithms for their recoveries. Due to the random selection of pixels strategy adopted for the DL in the C-SCD algorithm, the recovery of the Orange Perspex targets by the C-SCD is seen to fail as shown in Figure 7b. In contrast, these Orange Perspex targets have been successfully recovered by the proposed KMSCD, as shown in Figure 7c.

**Figure 8:**
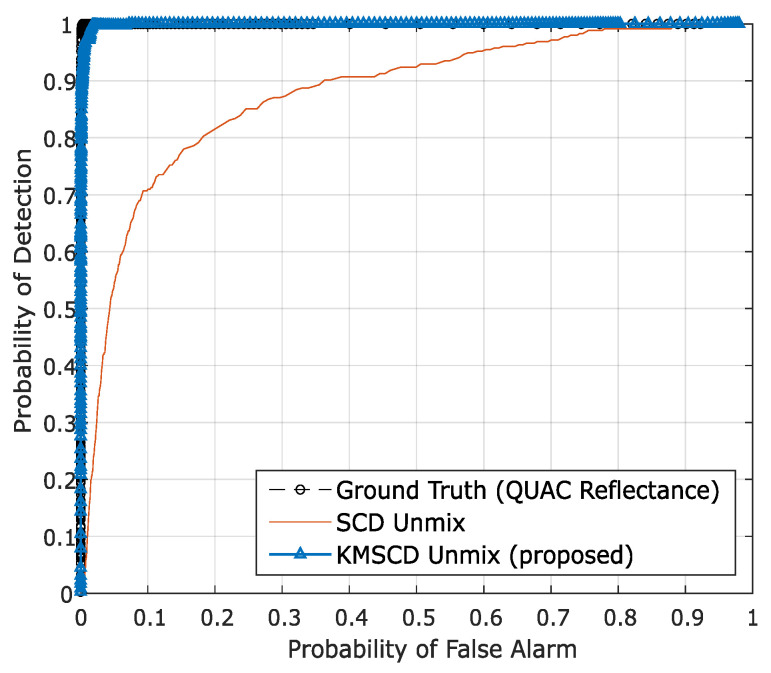
The receiver operating characteristic (ROC) for the detection of the Orange Perspex targets from the Selene Dual scene reconstructed by the C-SCD and KMSCD algorithms. The small orange targets are seen to be ~12% better detected from the KMSCD reconstructed scene.

To quantify the ability of the DL algorithms for the retrieval of these small targets, the receiver operating characteristic (ROC) for the detection of the Orange Perspex from the Selene Dual scene, reconstructed by C-SCD and the KMSCD for the same run shown previously with 50 EMs, are shown in Figure 8. It is seen quite clearly from the ROC curve that the Orange Perspex targets have been ~12% better detected by the KMSCD than that from the C-SCD data, as according to the conventional assessment method using the aura under the curve (AUC) metric for assessing the detection capability quantitatively.

#### 5.3.2. KMSCD for Scene Simulation Applications

**Figure 9:**
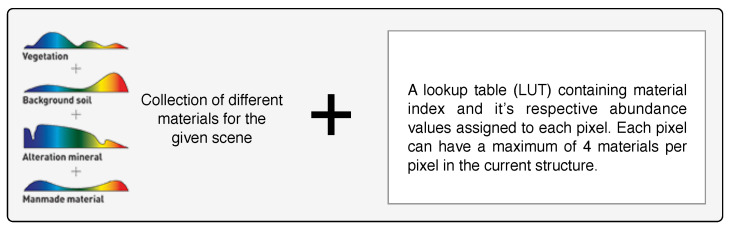
Structure of EM-abundance input used by a scene simulator.

Most HSI scene simulators, such as the commercial-off-the-shelf CameoSim package [31], impose a limited number of materials ($N_{mp}$) that can coexist within every pixel in the scene, shown in Figure 9. The consequence of this constraint is that it may affect the HSI reconstruction accuracy, and it is the purpose of this section to attempt to assess the side effect of this constraint. Two different ways to constraint the $N_{mp}$ have been implemented here: one is the use of the FNNOMP (i.e., the Algorithm 2, KMSCD+FNNOMP in Section 3), and the other employs the Texture Material Mapper (TMM) method (i.e., the KMSCD+TMM).

**Figure 10:**
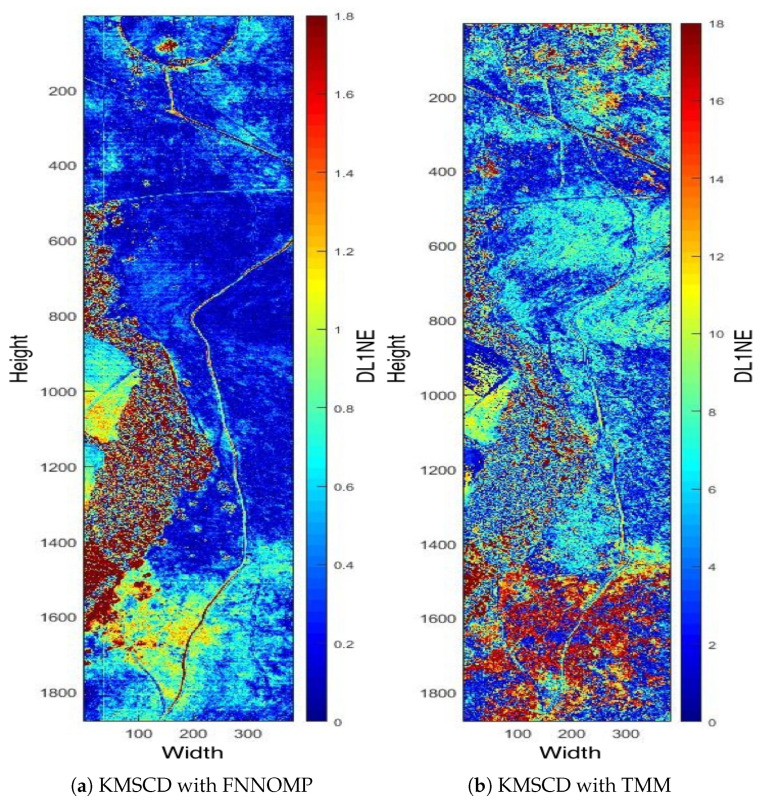
Selene H23 Dual scene by (**a**) the KMSCD+FNNOMP (Algorithm 2) and (**b**) the KMSCD+TMM. The mean errors of the entire map for panels (a,b) are 0.74% and 7.12%, respectively, showing the superiority of the FNNOMP over the TMM for constraining $N_{mp}$ to four materials per pixel.

**Figure 11:**
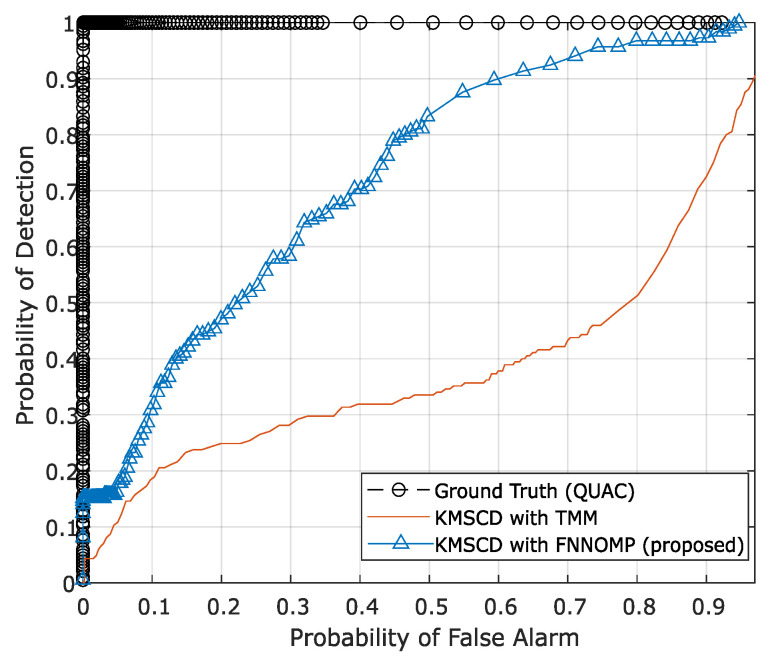
The ROC curve of Orange Perspex (OP) target material with adaptive cosine estimator (ACE). ACE detector that shows the better recovery of the trace materials (Orange Perspex) from the one reconstructed by the Algorithm 2 with an area under the curve (AUC) of 0.68, which is almost twice as that constrained by TMM (AUC = 0.37).

The TMM technique [26] has been used extensively in the HSI simulator (e.g., the CameoSim package) [32,33,34], which estimates the abundances by evaluating the inverse of the Euclidean distances of each EM with respect to the spectral characteristics of the mixed pixel, i.e., the test pixel. Note that the results that have been presented in Section 5.2.2 are the reconstructions that have been processed without the limitation on the $N_{mp}$. Figure 10 depicts the false-colour DL1NE map of the Selene Dual scene reconstructed by Algorithm 2 (Figure 10a) and the TMM (Figure 10b) using a maximum of four materials ($N_{mp}$) in every pixel. The mean DL1NE error of the whole scene for the reconstruction by Algorithm 2 is found to be 0.74%, which is more than 2 times higher than that of the Algorithm 1 (i.e., the KMSCD without $N_{mp}$ restriction). Furthermore, the mean DL1NE of the reconstruction that constrained by the TMM exhibits 7.12% error, which is ~10 orders of magnitude higher than that of Algorithm 2. Figure 11 plots the ROC for the detection of the Orange Perspex target from these two scenes by the ACE detector, and the AUC of the Algorithm 2 shows approximately twice as that of the one constrained by TMM. The combined results of these two figures may suggest that the proposed DL method using KMSCD and, together with the FNNOMP, will be more suitable for HSI scene simulators like the CameoSim as the material allocation method for practical HSI scene simulation applications.

## 6. Discussion and Conclusions

This paper proposes a simple yet effective method for improving the efficiency of sparse coding dictionary learning (DL), thereby the robustness and effectiveness of applications, which make use of compressive sensing (CS) technology, can be enhanced. CS is the technique which allows sparse signals to be decomposed into a sparse representation “a” of a dictionary $D_{u}$, and this dictionary $D_{u}$ has to be learnt (or trained) from a comprehensive data base. The dictionary, in theory, should encompass information and characteristics of all signals in the test dataset, and, in most cases, the dictionary is constituted from the data cloud of the test scene which is also known as the self-dictionary.

This paper proposes the construction of a concise and comprehensive dictionary by using the cluster centres of the input dataset, and then a greedy approach is adopted to learn all elements within this endmember dictionary. The proposed method consists of an unsupervised clustering algorithm (K-Means), and it is then coupled with an advanced sparse coding dictionary (SCD) method such as the basis pursuit algorithm (orthogonal matching pursuit OMP) for the dictionary learning. The effectiveness of the proposed KMSCD is illustrated through the reconstructions of several publicly available HSI scenes.

Before the performance of the proposed KMSCD method is assessed, this paper first outlines the practicality of using clustering method when the input data only consists of a few number of spectral bands. This must be investigated, as the multispectral image (MSI) has been commonly employed as the inputs for scene reconstructions. The result of Section 5.1 has indicated that the K-Means clustering works equally well as that of the all bands HSI input, even when the MSI scene contains <10 bands. Subsequently, the performance of the scene reconstruction by the proposed KMSCD has been shown to be almost twice as robust as the C-SCD over the test of the five repeated runs experiment (see Section 5.2.1). The capability of the scene reconstruction (i.e., reconstruction of background pixels) for the KMSCD has been shown to be ~40% better than that of the C-SCD in the reconstruction of the Selene dataset. Over the five data sets that have been employed in this study, it is seen from Table 2 that the proposed KMSCD is capable of reconstructing these scenes with mean accuracies approximately 20–500% better than all competing algorithms adopted in this work. With respect to the reconstruction efficiency of trace materials in the scene, the KMSCD is capable of ~12% improved recovery compared with the C-SCD (see Section 5.3.1). These results, together with the fast convergences in the KMSCD (see Section 4), have shown that the proposed DL that employs a simple clustering method for the construction of the dictionary enhances the scene reconstruction substantially.

Finally, the usefulness of the KMSCD is demonstrated for the scene simulation application when the number of materials coexists in a pixel is constrained to a maximum of four. In this experiment, the constraint is implemented by using the FNNOMP algorithm, and it is found that the constraint reduces the reconstruction accuracy of KMSCD by a factor of 2. However, this method achieves ~10 times better than that constrained by using the widely employed TMM algorithm. This may suggest that the proposed DL method using KMSCD and, together with the FNNOMP, will be more suitable to be the material allocation module of HSI scene simulators like the CameoSim package.

## Figures and Tables

**Table 1:** The dimensions of the hyperspectral scenes used in this paper.

Hyperspectral Images	Lines	Samples	Bands	Spectral Range (in μm)
Selene H23 VNIR	3752	1600	160	0.41 to 1
Selene H23 Dual	1876	380	448	0.41 to 2.5
Paso Robles-Monterey	5115	741	224	0.36 to 2.5
Virginia City 1807-1211	6349	320	178	0.4 to 2.45
Virginia City 1807-1220	6758	320	178	0.4 to 2.45
Virginia City 1807-1259	6904	320	178	0.4 to 2.45

**Table 2:** Mean DL1NE on various hyperspectral scenes.

Hyperspectral Images	Proposed	C-SCD Unmix	SD-SOMP	CoNMF	MVSA	VCA
Selene H23 VNIR	**1.249**	1.601	2.542	2.327	2.990	2.344
Selene H23 Dual	**0.282**	0.405	1.376	0.606	0.986	1.319
Paso Robles-Monterey	1.227	1.222	4.274	**0.768**	9.037	9.037
Virginia City 1807-1220	**0.054**	0.110	0.858	0.155	2.574	2.572
Virginia City 1807-1259	**0.061**	0.128	1.057	0.173	2.827	2.825
Mean error	**0.57**	0.69	2.02	0.81	3.68	3.62
± Std	±**0.61**	±0.68	±1.42	±0.89	±3.1	±3.08
Enhanced reconstruction accuracy						
over 5 datasets w.r.t. KMSCD		20.64%	251.79%	40.24%	540.93%	529.9%

First 1000 lines is used for training the dictionary with 50 atoms each for scenes Selene H23 VNIR, Selene H23 Dual and Paso Robles-Monterey. Reconstructed Virginia City images were trained on Virginia City 1807-1211.

**Table 3:** Mean Manhattan distance error on various hyperspectral scenes.

Hyperspectral Images	Proposed	SCD Unmix	SD-SOMP	CoNMF	MVSA	VCA
Selene H23 VNIR	**1.47**	1.6	2.65	2.29	2.77	2.83
Selene H23 Dual	**2.33**	2.51	3.94	2.69	3.63	5.46
Paso Robles-Monterey	**1.93**	1.99	7.37	**1.93**	15.79	15.79
Virginia City 1807-1220	**0.25**	0.3	0.94	**0.25**	0.84	0.99
Virginia City 1807-1259	**0.25**	0.28	0.98	**0.25**	0.82	0.99
Mean error	**1.24**	1.34	3.18	1.48	4.77	5.21
± Std	±**0.96**	±1.01	±2.66	±1.16	±6.28	±6.19
Enhanced reconstruction accuracy						
over 5 datasets w.r.t. KMSCD		7.22%	154.9%	18.94%	282.83%	318.4%

First 1000 lines is used for training the dictionary with 50 atoms each for scenes Selene H23 VNIR, Selene H23 Dual and Paso Robles-Monterey. Reconstructed Virginia City images were trained on Virginia City 1807-1211.

**Table 4:** Mean Manhattan distance error per band on various hyperspectral scenes.

Hyperspectral Images	Proposed	SCD Unmix	SD-SOMP	CoNMF	MVSA	VCA
Selene H23 VNIR	**9.2e-03**	1.0e-03	1.66e-02	1.43e-02	1.73e-02	1.77e-02
Selene H23 Dual	**5.2e-03**	5.6e-03	8.8e-02	6.0e-03	8.1e-03	1.22e-02
Paso Robles-Monterey	**8.6e-03**	8.9e-03	3.29e-02	**8.6e-03**	7.05e-02	7.05e-02
Virginia City 1807-1220	**1.4e-03**	1.7e-03	5.3e-03	**1.4e-03**	4.7e-03	5.6e-03
Virginia City 1807-1259	**1.4e-03**	1.6e-03	5.5e-03	**1.4e-03**	4.6e-03	5.6e-03
Mean error	**5.2e-03**	5.6e-03	1.38e-02	6.3e-03	2.1e-02	2.23e-02
± Std	±**3.8e-03**	±3.9e-03	±1.16e-02	±5.4e-03	±2.81e-02	±2.74e-02
Enhanced reconstruction accuracy						
over 5 datasets w.r.t. KMSCD		7.75%	167.83%	22.87%	307.75%	332.56%

First 1000 lines is used for training the dictionary with 50 atoms each for scenes Selene H23 VNIR, Selene H23 Dual and Paso Robles-Monterey. Reconstructed Virginia City images were trained on Virginia City 1807-1211.

## Abbreviations

The following abbreviations are used in this manuscript.

ACE	Adaptive Cosine Estimator
AUC	Area Under Curve
C-SCD	Classic Sparse Coding Dictionary
DL	Dictionary Learning
ED	Euclidean Distance
EM	Endmember
GSD	Ground Sampling Distance
KMSCD	K-Means Sparse Coding Dictionary
LUT	Lookup Table
ROC	Receiver Operating Characteristic
SCD	Sparse Coding Dictionary
HSI	Hyperspectral Image
MD	Manhattan Distance
MSI	Multispectral Image
ROC	Receiver Operating Characteristics
TMM	Texture Material Mapper

## References

[1] Yuen P.W., Richardson M. (2010). An introduction to hyperspectral imaging and its application for security, surveillance and target acquisition. Imaging Sci. J..

[2] Ward J.T. (2008). Realistic Texture in Simulated Thermal Infrared Imagery. Ph.D. Thesis.

[3] Pereira W., Richtsmeier S., Carr S., Kharabash S., Brady A. A comparison of MCScene and CameoSim simulations of a real scene. Proceedings of the 2014 6th Workshop on Hyperspectral Image and Signal Processing: Evolution in Remote Sensing (WHISPERS).

[4] Evans R. (2007). Modeling of SOC-700 Hyperspectral Imagery with the CAMEO-SIM Code. Proceedings of the 2007 Ground Systems Modeling, Validation & Testing Conference.

[5] James I., Richardson M., O’Keefe E. (2019). Comparison of empirical and predicted ultraviolet aircraft signatures. Opt. Eng..

[6] Li H., Shen H., Yuan Q., Zhang H., Zhang L., Zhang L. Quality improvement of hyperspectral remote sensing images: A technical overview. Proceedings of the 2016 8th Workshop on Hyperspectral Image and Signal Processing: Evolution in Remote Sensing (WHISPERS).

[7] Bioucas-Dias J.M., Plaza A., Dobigeon N., Parente M., Du Q., Gader P., Chanussot J. (2012). Hyperspectral Unmixing Overview: Geometrical, Statistical, and Sparse Regression-Based Approaches. IEEE J. Sel. Top. Appl. Earth Obs. Remote Sens..

[8] Charles A.S., Olshausen B.A., Rozell C.J. (2011). Learning Sparse Codes for Hyperspectral Imagery. IEEE J. Sel. Top. Signal Process..

[9] Han X., Yu J., Luo J., Sun W. (2019). Reconstruction From Multispectral to Hyperspectral Image Using Spectral Library-Based Dictionary Learning. IEEE Trans. Geosci. Remote Sens..

[10] Aharon M., Elad M., Bruckstein A. (2006). K-SVD: An algorithm for designing overcomplete dictionaries for sparse representation. IEEE Trans. Signal Process..

[11] Fu X., Ma W., Chan T., Bioucas-Dias J.M. (2015). Self-Dictionary Sparse Regression for Hyperspectral Unmixing: Greedy Pursuit and Pure Pixel Search Are Related. IEEE J. Sel. Top. Signal Process..

[12] Degerickx J., Okujeni A., Iordache M.D., Hermy M., Van der Linden S., Somers B. (2017). A Novel Spectral Library Pruning Technique for Spectral Unmixing of Urban Land Cover. Remote Sens..

[13] Fu X., Ma W., Bioucas-Dias J.M., Chan T. (2016). Semiblind Hyperspectral Unmixing in the Presence of Spectral Library Mismatches. IEEE Trans. Geosci. Remote Sens..

[14] Kapoor A., Singhal A. A comparative study of K-Means, K-Means++ and Fuzzy C-Means clustering algorithms. Proceedings of the 2017 3rd International Conference on Computational Intelligence Communication Technology (CICT).

[15] Haut J.M., Paoletti M., Plaza J., Plaza A. (2017). Cloud implementation of the K-means algorithm for hyperspectral image analysis. J. Supercomput..

[16] Liu Y., Guo Y., Li F., Xin L., Huang P. (2019). Sparse Dictionary Learning for Blind Hyperspectral Unmixing. IEEE Geosci. Remote Sens. Lett..

[17] Nascimento J.M.P., Dias J.M.B. (2005). Vertex component analysis: A fast algorithm to unmix hyperspectral data. IEEE Trans. Geosci. Remote Sens..

[18] Li J., Agathos A., Zaharie D., Bioucas-Dias J.M., Plaza A., Li X. (2015). Minimum Volume Simplex Analysis: A Fast Algorithm for Linear Hyperspectral Unmixing. IEEE Trans. Geosci. Remote Sens..

[19] Li J., Bioucas-Dias J.M., Plaza A., Liu L. (2016). Robust Collaborative Nonnegative Matrix Factorization for Hyperspectral Unmixing. IEEE Trans. Geosci. Remote Sens..

[20] Gao D., Hu Z., Ye R. (2018). Self-Dictionary Regression for Hyperspectral Image Super-Resolution. Remote Sens..

[21] Fang L., Li S., Kang X., Benediktsson J.A. (2015). Spectral–Spatial Classification of Hyperspectral Images With a Superpixel-Based Discriminative Sparse Model. IEEE Trans. Geosci. Remote Sens..

[22] Bao C., Ji H., Quan Y., Shen Z. (2016). Dictionary Learning for Sparse Coding: Algorithms and Convergence Analysis. IEEE Trans. Pattern Anal. Mach. Intell..

[23] Schnass K. (2018). Average Performance of Orthogonal Matching Pursuit (OMP) for Sparse Approximation. IEEE Signal Process. Lett..

[24] Yaghoobi M., Wu D., Davies M.E. (2015). Fast Non-Negative Orthogonal Matching Pursuit. IEEE Signal Process. Lett..

[25] Chang C. (2017). Adaptive Linear Spectral Mixture Analysis. IEEE Trans. Geosci. Remote Sens..

[26] Jacobsson J. (2005). Terrain Model Generation for the Infra Red Scene Simulation Software SensorVision(TM). Master’s Thesis.

[27] Piper J. A new dataset for analysis of hyperspectral target detection performance. Proceedings of the HSI 2014, Hyperspectral Imaging and Applications Conference.

[28] Bernstein L.S., Jin X., Gregor B., Adler-Golden S.M. (2012). Quick atmospheric correction code: Algorithm description and recent upgrades. Opt. Eng..

[29] Nasrabadi N.M. (2014). Hyperspectral Target Detection: An Overview of Current and Future Challenges. IEEE Signal Process. Mag..

[30] Basener W.F., Allen B., Bretney K. (2017). Geometry of statistical target detection. J. Appl. Remote Sens..

[31] Houlbrook A.W., Gilmore M.A., Moorhead I.R., Filbee D.R., Stroud C.A., Hutchings G., Kirk A. (2000). Scene simulation for camouflage assessment. Targets and Backgrounds VI: Characterization, Visualization, and the Detection Process.

[32] Sun K., Li Y., Gao J., Wang J., Wang J., Xie J., Ding N., Sun D., Guina M., Gong H., Niu Z., Lu J. (2014). Simulation system of airborne FLIR searcher. International Symposium on Optoelectronic Technology and Application 2014: Infrared Technology and Applications.

[33] AI Z., Zhang L., Zhang J. A practical method of texture segmentation and transformation for radar image simulation. Proceedings of the 2012 IEEE International Conference on Computer Science and Automation Engineering (CSAE).

[34] Zhou Q., Puschell J., Gong H., Cai Y., Lu J., Fei J. (2009). Dynamic scene simulation technology used for infrared seeker. International Symposium on Photoelectronic Detection and Imaging 2009: Advances in Infrared Imaging and Applications.

